# Diabetes and Renal Complications: An Overview on Pathophysiology, Biomarkers and Therapeutic Interventions

**DOI:** 10.3390/biomedicines12051098

**Published:** 2024-05-15

**Authors:** Rajesh Jha, Sara Lopez-Trevino, Haritha R. Kankanamalage, Jay C. Jha

**Affiliations:** 1 Kansas College of Osteopathic Medicine, Wichita, KS 67202, USA; rkjhadr@gmail.com; 2 Department of Diabetes, School of Translational Medicine, Monash University, Melbourne, VIC 3004, Australia

**Keywords:** diabetes, chronic kidney disease, albuminuria, inflammation, fibrosis, oxidative stress

## Abstract

Diabetic kidney disease (DKD) is a major microvascular complication of both type 1 and type 2 diabetes. DKD is characterised by injury to both glomerular and tubular compartments, leading to kidney dysfunction over time. It is one of the most common causes of chronic kidney disease (CKD) and end-stage renal disease (ESRD). Persistent high blood glucose levels can damage the small blood vessels in the kidneys, impairing their ability to filter waste and fluids from the blood effectively. Other factors like high blood pressure (hypertension), genetics, and lifestyle habits can also contribute to the development and progression of DKD. The key features of renal complications of diabetes include morphological and functional alterations to renal glomeruli and tubules leading to mesangial expansion, glomerulosclerosis, homogenous thickening of the glomerular basement membrane (GBM), albuminuria, tubulointerstitial fibrosis and progressive decline in renal function. In advanced stages, DKD may require treatments such as dialysis or kidney transplant to sustain life. Therefore, early detection and proactive management of diabetes and its complications are crucial in preventing DKD and preserving kidney function.

## 1. Introduction

Diabetic kidney disease (DKD) poses a heavy global burden as the leading cause of end-stage renal disease (ESRD) [1,2,3]. DKD develops in approximately 30–40% of patients with diabetes and as of 2021, about 537 million (11% of the global population) people worldwide had diabetes which is expected to increase to 783 million by 2025 [4]. Thus, in parallel, the global prevalence of DKD and its associated long-term complications are also expected to increase [1]. Despite the use and emergence of many therapeutic approaches for the treatment of DKD such as renin–angiotensin system blockers (ACE inhibitors and angiotensin receptor blockers), mineralocorticoid receptor blockers and sodium–glucose cotransporter 2 (SGLT2) inhibitors, there is still no cure and many patients still progress to end-stage renal disease (ESRD) [5,6,7]. At this stage of kidney failure, many patients require dialysis or kidney transplantation, show increased morbidity and mortality, reduced quality of life as well as placing a significant burden on healthcare systems worldwide, thus emphasising the need for more effective therapeutic approaches as well as early diagnosis of DKD for the risk prediction [7]. In line with this, here, we provide an update on the pathophysiology of renal complications of diabetes and associated biomarkers as well as current and potential therapeutic interventions to combat the progression and treatment of this devastating disease.

## 2. Pathophysiology of Diabetic Kidney Disease

**Figure 1:**
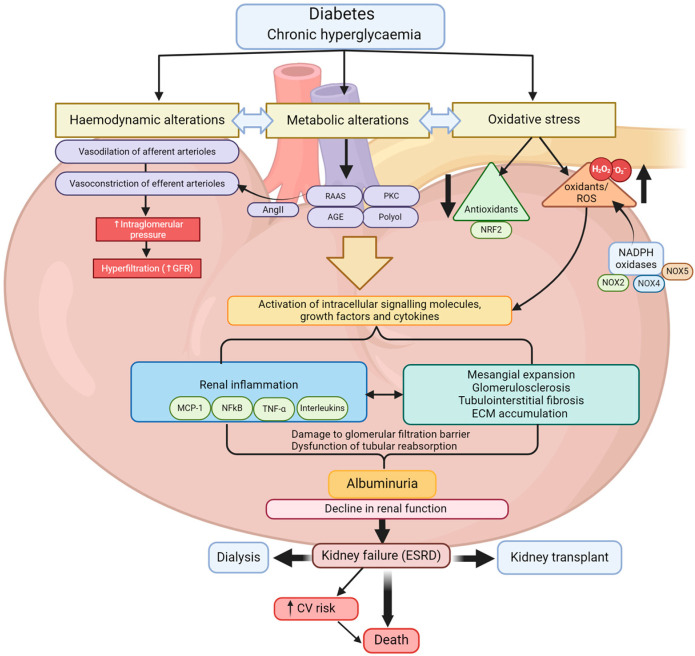
Schematic outlining the major pathways involved in the pathogenesis of DKD.

The pathophysiology of DKD is multifactorial in that the diabetes-induced hyperglycaemia drives various pathological pathways within the kidney which can be broadly characterised as haemodynamic, metabolic, inflammatory, fibrotic and oxidative stress as the major determinant of this pathological process. There is a substantial overlap and a dynamic interplay among these pathways, which occur in multiple renal compartments such as glomerular, vasculature, tubular and interstitium that drive the pathogenesis of DKD. The key features of diabetes-mediated renal complications include morphological and functional alterations to renal glomeruli and tubules leading to mesangial expansion, glomerulosclerosis, homogenous thickening of the glomerular basement membrane (GBM), albuminuria, tubulointerstitial fibrosis and progressive decline in renal function (Figure 1).

### 2.1. Haemodynamic Changes

Diabetes-mediated chronic hyperglycaemia, which causes an increase in the osmolarity within glomerular capillaries leads to an increase in glomerular pressure and dilation of afferent arterioles, causing glomerular hyperfiltration, resulting in increased glomerular filtration rate [8,9]. These haemodynamic alterations also initiate and activate several vasoactive systems within the kidneys that further contribute to the progression of DKD [10,11]. These include the activation of the renin–angiotensin–aldosterone system (RAAS), protein kinase C (PKC), polyol and advanced glycation end product (AGE) dependent pathways as well as the more recently explored pro-oxidant enzyme NADPH oxidases [12,13,14,15,16]. In particular, the increase in angiotensin II which is a key effector hormone of the RAAS stimulates the vasoconstriction of the efferent arteriole, further increasing the intraglomerular pressure and subsequent hyperfiltration [17,18,19]. The resulting increase in glomerular filtration rate and glomerular capillary pressure leads to glomerular and tubular injury. Furthermore, angiotensin II stimulates the expression of various pro-inflammatory and profibrotic mediators by direct cellular effects or indirectly via the barotrauma within glomerular capillaries, driving the key pathologies of inflammation and fibrosis in DKD [20]. Moreover, in physiological conditions, the kidney ensures glucose homeostasis via the reabsorption of filtered glucose in the proximal tubules [20,21,22]. This is primarily mediated by sodium–glucose cotranspoter-2 (SGLT2) expressed on the luminal surface of proximal tubular epithelial cells which is responsible for approximately 90% of glucose reabsorption [20,21,22]. However, in diabetic conditions, excessive glucose filtration through the glomeruli occurs causing glycosuria as a common symptom. In this state, the SGLT2 expression and activity in the proximal tubules increase as an adaptation to replenish the filtered glucose from the urine [21]. However, the increased glucose reabsorption augments hyperglycaemia. The increase in proximal sodium reabsorption due to enhanced SGLT2 activity results in the decrease in sodium delivery to the cells of macula densa via the tubule-glomerular feedback mechanism and consequently reducing adenosine production which is a potent vasoconstrictor [21], thus, leading to the vasodilation of the afferent arteriole, further exacerbating hyperfiltration.

### 2.2. Metabolic Pathways and Oxidative Stress

Oxidative stress plays a critical role in the initiation and progression of DKD [14]. Chronic hyperglycaemia induces oxidative stress via enhanced reactive oxygen species (ROS) production and diminished antioxidant defence in DKD [14]. Chronic hyperglycaemia disrupts the homeostatic balance between pro-oxidant and antioxidant pathways whereby there is an upregulation of pro-oxidant enzyme-derived ROS formation and an accompanied reduction in antioxidants, causing oxidative stress within the kidneys [22,23,24,25]. Recent accumulating evidence demonstrates the overproduction of intrarenal ROS in diabetes and subsequent haemodynamic alterations and metabolic changes as a key mediator and a common denominator of the pathways leading to disrupted renal function and pathological structural changes in DKD [6,17,22]. In particular, alterations to metabolic pathways including the protein kinase C, polyol, hexosamine and the accumulation of advanced glycation end products (AGEs) contribute to the progression of DKD, especially via enhanced ROS formation in the kidneys. Furthermore, more recent findings highlight that the pro-oxidant enzymes, NADPH (Nicotinamide adenine dinucleotide phosphate) oxidases, also known as NOXs are a major source of ROS within the kidneys and the overproduction of ROS by them is a key contributor in driving the pathological pathways leading to renal damage [14]. The increase in ROS formation via altered metabolic pathways and directly via NADPH oxidases eventually leads to the pathological changes characterising DKD including renal inflammation, fibrosis and albuminuria [25,26,27]. Among the many pathways implicated in ROS production, recent studies highlight the NOXs, particularly NOX4 and NOX5 as the most important contributors [12,28,29].

The role of NOXs in DKD was supported by the finding of increased expression of the pro-oxidant NADPH oxidase subunits which were observed on the gene and protein level in renal cells including podocytes, mesangial and endothelial cells with concomitant downregulation of antioxidant enzymes (e.g., glutathione peroxidase) when exposed to high glucose conditions [30,31]. The high glucose-induced biochemical changes eventually resulted in increased ROS production leading to hydrogen peroxide accumulation in the extracellular space [24]. There are also other sources of ROS, including mitochondrial ROS but it is considered that NADPH oxidases are the major source of ROS in long-term diabetic kidney disease [14]. The overproduction of ROS due to hyperglycaemia stimulates the activation and recruitment of intracellular signalling molecules including cytokines, growth factors and transcription factors which drive the pathological pathways of renal inflammation and fibrosis. NRF2 (nuclear factor erythroid 2-related factor 2) is a transcription factor that plays a crucial role in cellular defence against oxidative stress and inflammation by regulating the expression of antioxidant and detoxifying enzymes. Kelch-like ECH-associated protein 1 (Keap1) is a negative regulator of NRF2. In a preclinical model of DKD, Nrf2 expression is found to be downregulated in association with enhanced ROS production via upregulation of Keap1 leading to depletion in the antioxidant defence mechanism [32].

### 2.3. Renal Inflammation

In DKD, inflammation plays an important role. It is driven by multiple pathways including oxidative stress, activation of transcription pathways such as JAK/STAT and transcription factors including nuclear factor κB (NF-κB) and the activation of pro-inflammatory cytokines. While in physiological conditions, reactive oxygen species play a role in immune defence in various immune cell lines, the overproduction of ROS in diabetic conditions stimulates the production of numerous inflammatory cytokines and thus subsequent recruitment of multiple inflammatory cell types. Various transcription pathways are also activated such as the JAK/STAT signal transduction that activates NF-κB (nuclear factor-kappa B) which is recognised as a key transcription factor in regulating cytokine production leading to the transcription of cytokines, chemokines and adhesion molecules, driving multiple inflammatory processes. NF-κB has been observed to be significantly enhanced in diabetic conditions as opposed to non-diabetic conditions [33,34]. In high glucose conditions, this transcription factor has been shown to be activated within mesangial cells through the increased activity of PKC and ROS and also indicated that it may play a role in the upregulation of monocyte chemoattractant protein-1 (MCP-1) both at the gene and protein levels [35].

Cytokines which are polypeptide signalling molecules are part of the innate immune response and in DKD both activation of circulating cytokines and the production of cytokines by inflammatory cells as well as intrinsic renal cells (such as tubular, glomerular, mesangial and endothelial cells) is elevated [36]. The main cytokines involved in renal inflammation include IL-1, IL-6, IL-18 and tumour necrosis factor-α (TNF-α) as these have been found to be upregulated in renal biopsies of patients with DKD [36]. Chemokines which are a subgroup of cytokines function as chemoattractant molecules that recruit inflammatory cells from the vasculature into renal structures, augmenting renal inflammation. In particular, the release of the MCP-1 at the early stages of disease progression induces monocyte and macrophage infiltration into the renal parenchyma. These recruited monocytes and macrophages further augment the inflammation via cytokine production, especially MCP-1 production and the subsequent recruitment of pro-inflammatory cells. MCP-1 expression has been observed to be upregulated in experimental models of diabetes, especially within glomeruli and tubulointerstitium [28]. MCP-1 levels have also been identified to be significantly increased in renal biopsies of patients with DKD along with enhanced urinary MCP-1 excretion compared to non-diabetic individuals [37]. MCP-1 is known to play a multifaceted role in the pathogenesis of DKD, especially via promoting inflammation, inducing fibrotic responses as well and driving haemodynamic alterations leading to albuminuria. Furthermore, the release of the pleiotropic cytokine TNF-α from an early stage of disease progression is involved in the induction of the inflammatory response, immune modulation and apoptotic cell death [38]. It is also known to be associated with haemodynamic alterations, particularly in increasing vascular endothelium permeability as well as enhancing oxidative stress in DKD. In addition, the cytokines, IL-1, IL-6, and IL-8 also contribute to the pathogenesis of DKD via a variety of mechanisms [39]. IL-1 induces the production and release of prostaglandin E and phospholipase A2, particularly in mesangial cells which increases glomerular hyperperfusion by enhancing endothelial cell permeability [40]. IL-6 is involved in neutrophil infiltration into the tubulointerstitium, podocyte hypertrophy and glomerular basement membrane (GBM) thickening [41]. In addition, IL-18 mediates apoptosis in renal cells and increases the release of INF-γ and adhesion molecule expression [39,42].

### 2.4. Renal Fibrosis

Renal fibrosis is recognised as a key hallmark in the progression of DKD, particularly in driving renal structural changes especially extracellular matrix (ECM) accumulation causing renal injury. High glucose conditions have been found to enhance the activation of profibrotic growth factors including connective tissue growth factor (CTGF) as well as transforming growth factor-β (TGF-β) which recruits and activates extracellular matrix-producing cells. The activation of such growth factors induces intrinsic renal cells, particularly epithelial and endothelial cells to undergo epithelial-to-mesenchymal transition (EMT) and endothelial-to-mesenchymal transition (EndoMT), forming myofibroblasts, which secrete ECM proteins such as collagen I, III and IV as well as fibronectin causing renal fibrosis and eventually glomerulosclerosis [43]. Fibroblasts and pericytes are also activated in the hyperglycaemic state to form myofibroblasts, thus accelerating renal injury. The occurrence of renal fibrosis is characterised by structural changes including ECM protein accumulation, renal hypertrophy, thickened glomerular basement membrane, mesangial expansion, podocyte damage and loss as well as tubulointerstitial fibrosis [44]. Mesangial cells are especially known to be involved in the development and increased deposition of ECM proteins such as collagen IV and fibronectin [12]. These cells undergo hypertrophy and activation as a result of oxidative stress-mediated activation of protein kinase B and transforming growth factor beta 1 (TGF-β1) in diabetic conditions [45]. Increased mesangial matrix formation results in enhanced mesangial expansion, eventually leading to glomerulosclerosis [12]. Previous findings also demonstrate that activated vasoactive agents such as angiotensin II and endothelin are involved in enhancing TGF-β expression in experimental models, both in vitro and in vivo [14]. Recent studies highlight that enhanced ROS formation plays a critical role in driving pro-fibrotic pathways, especially by stimulating growth factors and cytokines. Such pro-fibrotic pathways cause irreversible structural damage to renal parenchyma causing sclerosis and accelerating renal disease in diabetes.

### 2.5. Glomerular Filtration Barrier Alteration and Albuminuria

Albuminuria is recognised as an early predictor for the progression of DKD. The variety of pathological mechanisms including oxidative stress, renal inflammation, fibrosis and haemodynamic alterations lead to the damage of the highly regulated glomerular filtration barrier, compromising the filtration process. Damage to the glomerular basement membrane, particularly to the podocytes which uphold the structural integrity of the filtration barrier is an early feature in the progression of DKD [46]. Podocyte injury includes foot process effacement as well as podocyte detachment and depletion, which can lead to glomerular endothelial cell dysfunction [31,47]. Damage to these epithelial renal cells involved in filtration results in the leakage of protein through the filtration barrier of the glomeruli causing increased albumin and protein excretion in the urine, known as albuminuria and proteinuria [48]. As DKD progresses, initially seen microalbuminuria (albumin excretion rate of 30-299 mg/g creatinine) will progress to macroalbuminuria (≥ 300 mg/g creatinine) [49,50,51]. This is indeed a hallmark of DKD, and murine models have shown significantly elevated albuminuria in diabetic mice compared to non-diabetic control mice [31].

### 2.6. Role of Renal Biopsy in DKD

Renal biopsy plays a crucial role in the diagnosis and management of DKD. It provides evidence of kidney damage caused by diabetes, distinguishing it from other forms of kidney disease that may occur concurrently with diabetes. It helps in assessing the severity of kidney damage caused by diabetes. This information is crucial for guiding treatment decisions and predicting prognosis. Renal biopsy allows for the identification of specific pathological features of DKD, such as glomerular basement membrane thickening, mesangial expansion, and nodular glomerulosclerosis (Kimmelstiel–Wilson nodules) [52,53]. These features aid in understanding the underlying mechanisms of kidney damage in DKD. The information obtained from renal biopsy can guide treatment planning, including the selection of appropriate medications and interventions to slow the progression of DKD and manage complications.

Limitations of Renal Biopsy in DKD: Renal biopsy samples only a small portion of kidney tissue, leading to sampling variability that may not accurately reflect the full extent of kidney damage. This invasive procedure poses risks such as bleeding, infection, and injury, especially in diabetic patients with comorbidities. Interpretation challenges arise due to overlapping features with other kidney diseases, complicating diagnosis. In some cases, biopsy findings may not alter treatment decisions, potentially outweighing its benefits. Despite these limitations, renal biopsy remains an important tool in the management of DKD, particularly in cases where the diagnosis is uncertain, or when additional information is needed to guide treatment decisions. It should be performed judiciously, weighing the potential risks against the expected benefits on a case-by-case basis.

## 3. Biomarkers of Renal Complications of Diabetes

Biomarkers play a crucial role in the diagnosis and prognosis of DKD including monitoring the response to an intervention. Biomarkers provide valuable insights into the progression of the disease and help clinicians tailor treatment strategies [54]. Glomerular filtration rate (GFR) measures the rate at which the kidneys filter waste from the blood. In DKD, GFR declines as kidney function deteriorates. Estimated glomerular filtration rate (eGFR), calculated using serum creatinine levels, age, sex, and race, is a widely used biomarker to assess kidney function. A decline in eGFR over time indicates progressive kidney damage in DKD [55]. In addition to eGFR, the following are the preclinically and clinically validated biomarkers for DKD. The key characteristics of biomarkers of renal complications of diabetes are listed in Table 1.

### 3.1. Albuminuria (Proteinuria)

With the progression of kidney damage in diabetes, specifically glomerular injury, there is a loss of protein in the urine as a consequence of the glomerular filtration barrier losing its size and charge-selective properties [56]. Albumin is the most abundant protein found in the blood, and its presence in urine (albuminuria) is one of the earliest signs of kidney damage in diabetes. Increased urinary excretion of albumin, even in small amounts, is a hallmark of DKD. The level of albuminuria is positively correlated with DKD progression and is classified mainly into two categories, microalbuminuria with a concentration of 30–300 mg/day and macroalbuminuria with concentrations higher than 300 mg/day [57]. Quantifying the amount of albumin in urine through urine albumin-to-creatinine ratio (UACR) or urine protein-to-creatinine ratio (UPCR) is a standard method for assessing kidney damage in diabetes, specifically tracking the decline in estimated glomerular filtration rate (eGFR) [58,59].

### 3.2. Glycated Albumin

Chronic hyperglycaemia, one of the defining features of diabetes, induces functional and structural changes in serum proteins via increasing non-enzymatic glycation [60]. Glycated albumin offers a short-term measurement of glucose levels in serum and has been suggested as an alternative test for haematological conditions in which haemoglobin is altered [61]. Furthermore, glycated albumin has been shown to be a good predictor of chronic complications and a useful complement test when traditional tests are unavailable [62,63]. The most widely used technique to diagnose diabetes includes the assessment of the glycated haemoglobin (HbA1c), which can be used as a predictor of diabetes and future complications with high specificity [58,63,64].

### 3.3. Transferrin

There is an increasing amount of evidence that relates iron abnormalities to diabetes and the development of chronic complications [65]. A study by Cho et al. found that iron imbalances were associated with a higher mortality risk among people with diabetes [66]. It has been suggested that imbalances of iron homeostasis create iron stores, which can be related to DKD by causing excessive inflammation, oxidative stress, lipid peroxidation, and organelle damage, especially in the mitochondria and lysosomes [67,68,69]. Transferrin possesses a greater molecular weight than albumin, 76 and 66 kDa, respectively, however, its less anionic properties and molecular shape allow an easier filtration than that of albumin through the glomerular basement membrane [70,71,72]. Transferrinuria is considered as an earlier, sensitive and non-invasive diagnostic marker of DKD [73].

### 3.4. Adiponectin

Adiponectin is an adipocyte-derived hormone, that has been suggested to play a role as a biomarker and potential therapeutic target for mitigating diabetic complications including DKD [74,75]. Furthermore, adiponectin possesses anti-inflammatory, anti-oxidative, and vasoprotective properties as well as protects against insulin resistance in diabetes [76]. Lower levels of serum and higher levels of urinary adiponectin have been observed in individuals with diabetes when compared to non-diabetic subjects [77]. Therefore, adiponectin has been proposed as a potential biomarker for DKD risk prediction and monitoring of the disease progression. However, its utility as a standalone biomarker remains uncertain due to the variability in adiponectin levels and its complex interactions with other factors involved in DKD pathogenesis. Additional research is needed to elucidate the mechanisms underlying adiponectin’s effects on kidney function and its potential as a biomarker for DKD.

### 3.5. Ceruloplasmin

Ceruloplasmin is a multifunctional glycoprotein primarily known for its role in copper metabolism, but it also acts as an acute-phase reactant and antioxidant. In the context of DKD, ceruloplasmin has been studied as a potential biomarker due to its involvement in various physiological processes, including inflammation and oxidative stress, both of which play significant roles in the pathogenesis of DKD [78]. Serum ceruloplasmin is elevated, in diabetic patients, regardless of the type of diabetes [79]. Ceruloplasmin has been demonstrated to be anti-inflammatory due to its interactions with proteins such as lactoferrin and myeloperoxidase that reduce oxidative stress [80,81]. As serum ceruloplasmin has been correlated with HbA1c levels, it has been suggested as a marker to track glycaemic index and inflammation status in DKD [82]. While ceruloplasmin shows promise as a biomarker for DKD due to its involvement in inflammation, oxidative stress, and copper metabolism, its utility in clinical practice remains to be fully elucidated. Additional studies are needed to evaluate its sensitivity, specificity, and predictive value as a biomarker for DKD diagnosis, prognosis, and monitoring.

### 3.6. Laminin

Laminin is a major component of the extracellular matrix (ECM) in the basement membranes of various tissues, including the kidneys. In DKD, alterations in the expression and distribution of laminin in the renal ECM and within the GBM have been implicated in the development of glomerular damage and proteinuria in DKD [83,84]. Moreover, the laminin subunits LAMC1 and LAMB1 are associated with immune cells and are thought to promote inflammation in DKD [85]. Studies have investigated the utility of laminin and its fragments as biomarkers for DKD. Increased urinary excretion of laminin fragments, such as laminin alpha-5 chain fragment (C16), has been observed in individuals with DKD compared to those without kidney disease, suggesting a potential role for laminin as a biomarker of DKD as well as an early diagnostic marker of mesangial matrix expansion [83,84,85,86]. Elevated levels of laminin fragments in urine may reflect increased turnover of basement membrane proteins and ongoing renal injury in DKD. Additionally, increased levels of urinary laminin fragments have been associated with progressive decline in renal function and adverse outcomes in DKD, suggesting potential prognostic value for these biomarkers in predicting disease progression and response to therapy. While laminin and its fragments show promise as biomarkers of DKD, further research is needed to validate their clinical utility, including their sensitivity, specificity, and predictive value for diagnosing and monitoring DKD progression. Additionally, standardisation of assays and protocols for measuring laminin biomarkers is necessary to facilitate their implementation in clinical practice.

### 3.7. Podocyte Number and Podocyte-Specific Proteins

The podocyte is one of the major components of the glomerular basement membrane, which is involved in the filtration of metabolic wastes [87]. Diabetes-associated chronic podocyte injury can cause podocyte dedifferentiation, podocyte death, or/and podocyte detachment, leading to podocyte loss in urine [88,89]. Podocytes have been found to be absent in urine samples of healthy individuals, whereas their number increased from microalbuminuria to macroalbuminuria in individuals with diabetes [90]. Furthermore, mRNA levels of podocyte-related proteins such as nephrin, podocalyxin, synaptopodin, podocin, WT-1 and α-actin-4 were found to be elevated in DKD [91,92,93]. These podocyte-related proteins are considered to be potential diagnostic markers for early kidney injury [92,93,94].

### 3.8. NGAL

Neutrophil gelatinase-associated lipocalin (NGAL) is a small protein that is expressed by various cell types, including neutrophils, renal tubular cells, and epithelial cells. NGAL is released by damaged kidney tubular cells in response to injury or inflammation [95,96]. In DKD, increased renal expression and elevated levels of NGAL in urine or blood have been associated with albuminuria, decreased eGFR, and histological markers of kidney damage, such as tubulointerstitial fibrosis and glomerulosclerosis, suggesting that NGAL may serve as a marker of disease severity and progression in DKD [97,98,99]. Overall, NGAL shows promise as a biomarker for early detection, monitoring, risk stratification, and treatment response assessment in DKD [100].

### 3.9. NAG

N-acetyl-β-D-glucosaminidase (NAG) is an enzyme primarily found in the lysosomes of renal tubular cells. It plays a role in the breakdown of complex carbohydrates within the lysosomes and increased NAG activity in urine is indicative of tubular injury, as the enzyme leaks into the urine when tubular cells are damaged or stressed [101]. Therefore, urinary NAG levels can serve as a marker of tubular injury in DKD. It has been proposed as an early diagnostic biomarker of DKD since its production is independent of albuminuria and has been found to be in higher levels in diabetic normoalbuminuric patients [102]. Elevated urinary NAG levels have been associated with an increased risk of renal function decline and adverse renal events in individuals with DKD, independent of traditional risk factors in both Type 1 and Type 2 diabetes [103,104,105]. Reductions in urinary NAG levels following interventions aimed at improving glycaemic control, blood pressure management, and renoprotective therapies have been reported in individuals with DKD. This suggests that urinary NAG levels may serve as a marker of treatment efficacy and renal recovery in DKD.

### 3.10. KIM-1

Kidney injury molecule (KIM)-1 is an immunoglobulin superfamily protein expressed at very low levels in healthy kidneys but is markedly upregulated in renal tubular epithelial cells in response to injury. KIM-1 is considered a sensitive biomarker of acute kidney injury (AKI) and chronic kidney disease progression, including DKD. Elevated levels of KIM-1 in urine or blood may indicate tubular injury and ongoing kidney damage in DKD [106]. Elevated levels of KIM-1 have been observed in individuals with DKD compared to those without kidney disease, and KIM-1 levels tend to correlate with the severity of renal impairment and albuminuria [107]. Furthermore, an increased level of serum KIM-1 has been shown to predict the progression of type 1 diabetic patients to ESRD, suggesting that KIM-1 may serve as a marker of disease severity and progression in DKD [108]. In addition, KIM-1 identifies apoptotic cells and induces phagocytosis by directing them to lysosomes [109]. Moreover, KIM-1 promotes the uptake of fatty acids by proximal tubular cells, promoting inflammation and fibrosis, contributing to the progression of DKD and potentially representing a therapeutic target [110].

### 3.11. Cystatin C

Serum cystatin C shows promise as a biomarker for DKD due to its sensitivity to changes in glomerular filtration rate (GFR) and its potential to detect early kidney dysfunction [111]. However, its utility in clinical practice for DKD diagnosis and monitoring remains under investigation. Some limitations include variability in cystatin C levels due to factors like age, gender, and inflammation, as well as the need for standardised assays and reference ranges. Despite these challenges, serum cystatin C holds potential as a complementary biomarker alongside traditional markers like serum creatinine, offering valuable insights into DKD progression and response to treatment. Ongoing research aims to clarify its role and optimise its clinical application in DKD management.

### 3.12. YKL-40, TNFR1, and TNFR2

YKL-40, TNFR1, and TNFR2 have emerged as potential biomarkers for diabetic kidney disease, reflecting underlying inflammation and tissue damage in the kidneys. YKL-40, also known as chitinase-3-like protein 1, is associated with fibrosis and inflammation in DKD progression. TNFR1 and TNFR2 are receptors involved in the tumour necrosis factor (TNF) signalling pathway, implicated in inflammatory responses in DKD [112].

### 3.13. α1- and β2-Microglobulin

α1- and β2-Microglobulin are low molecular weight molecules that under normal conditions are almost completely reabsorbed by proximal tubule cells. However, upon decreased renal function as seen in DKD, their concentration increases in urine [113]. High levels of α1- and β2-Microglobulin were found in type 2 diabetic patients [114,115]. However, their diagnostic use has been proposed to be performed in combination with other biomarkers such as NAG and microalbumin since α1- and β2-microglobulin have not been efficient for predicting DKD progression [115,116].

### 3.14. Fatty-Acid-Binding Protein

Fatty-acid-binding proteins (FABPs) are a family of small cytoplasmic proteins that are involved in the transport and metabolism of fatty acids. Two types of FABPs are found in the kidneys, Liver-type fatty acid binding protein (L-FABP) and heart-type fatty acid binding protein (H-FABP). L-FABP is expressed in the proximal tubules whereas H-FABP in the distal tubules [117]. During renal tubular injury, such as that seen in DKD, FABPs are released into the urine due to damage to the tubular epithelial cells. Therefore, increased levels of urinary FABPs, particularly L-FABP and H-FABP, have been observed in DKD patients, indicating tubular injury and thus represent potential biomarkers [118]. In addition, L-FABP has been investigated as not only early biomarkers of kidney damage but also a potential therapeutic target in DKD [119].

### 3.15. RBP4

Retinol-binding protein 4 (RBP4) is a carrier protein that transports retinol (vitamin A) in the bloodstream. It has garnered attention as a potential biomarker in various metabolic disorders, including DKD [120]. Elevated serum levels of RBP4 have been observed in individuals with insulin resistance and type 2 diabetes. Elevated RBP4 levels have been associated with markers of inflammation and oxidative stress in individuals with DKD, suggesting that RBP4 may contribute to the pathogenesis of kidney damage in diabetes. Indeed, it has been suggested that RBP4 may contribute to DKD by decreasing insulin sensitivity in both type 2 diabetes and obesity by promoting a pro-inflammatory response in adipose tissue [121,122,123]. In a meta-analysis, it was found that RBP4 level increased with an increase in albumin-to-creatinine ratio and decreased GFR [124]. While RBP4 shows promise as a biomarker for DKD, further research is needed to validate its clinical utility, elucidate its role in the pathogenesis of DKD, and determine its potential as a target for therapeutic interventions.

### 3.16. Biomarkers of Inflammation and Fibrosis

Inflammatory markers such as C-reactive protein (CRP), tumour necrosis factor-alpha (TNF-α), interleukin-6 (IL-6) and chemoattractant protein-1 (MCP-1) as well as fibrotic markers including transforming growth factor-beta (TGF-β) and collagens are associated with the inflammatory and fibrotic processes underlying DKD progression [125,126]. Elevated levels of these biomarkers in blood or urine may indicate ongoing kidney damage and inflammation in DKD. It has been demonstrated that high glucose increases the production of collagen type IV via activation of the endogenous TGF-β pathway [127]. Elevated urinary excretion of collagen type IV has been found in patients with diabetes and correlates with extracellular matrix expansion [128,129]. Genome-wide association studies performed in individuals with type 1 diabetes have identified that the most prominent protective variant against DKD is a missense mutation in the gene COL4A3, however, further studies are required to elucidate its role in this disease [130]. A significant association between TNF and eGFR was observed in individuals with type 2 diabetes, potentially serving as indicators of disease progression [131,132,133]. Elevated levels of serum and urinary MCP-1 and IL-6 in DKD are associated with enhanced ACR and a decline in eGFR [134,135,136,137,138].

### 3.17. Biomarkers of Oxidative Stress

Oxidative stress has been identified as a major contributor to the pathogenesis and disease progression of DKD. Due to their short half-live, ROS are not suitable biomarkers, however, modifications induced by ROS such as lipid peroxidation, protein oxidation, and nucleic acid oxidation, are easier to detect and measure. Therefore, these modified products are considered biomarkers of oxidative stress in DKD which include malondialdehyde (MDA), F2-isoprostanes, advanced glycation end products (AGEs) and 8-hydrox-2’-deoxyguanosine (8-OHdG) [139]. These aforementioned oxidative stress markers have been shown to be elevated in both the plasma and urine of individuals with DKD when compared to individuals without renal complications and diabetes [140,141,142,143].

### 3.18. Novel Omics Approaches in DKD

Recently, omics approaches, including transcriptomics, proteomics, and metabolomics, have been utilised to identify genes and their products associated with diseases. In 2010, the urinary CKD273 test was developed using proteomics, marking a pioneering discovery in DKD biomarker diagnostics [144]. This test has demonstrated efficacy in predicting the risk of CKD development. To date, over 2500 genes and 650 proteins have been linked to DKD. Notably, collagen gene COL and its fragments have consistently emerged as significant in multiple studies [145,146,147]. Recent GWAS studies revealed that approximately half of these 2519 genes were upregulated and the other half downregulated in DKD [146]. At the protein level, out of 678 identified proteins, 279 were upregulated while the remainder were downregulated in DKD [146]. The integration of omics approaches shows promise in identifying new therapeutic targets and biomarkers for DKD.

### 3.19. Limitations in the Use of New Biomarkers

The integration of new biomarkers in diagnosing and managing chronic kidney disease (CKD) including DKD promises more accurate assessment and personalised treatments. However, limitations exist. Many lack validations across diverse populations and settings, requiring standardised assays for reliability. Cost and expertise constraints may hinder widespread adoption. Uncertainty surrounds certain biomarkers’ clinical significance and interpretation complexities when considering multiple biomarkers simultaneously. Ethical concerns, CKD’s heterogeneous nature, and confounding factors like comorbidities further complicate biomarker use. While some aid in risk prediction, accurately diagnosing CKD stages remains challenging. Individual biomarker variability necessitates personalised approaches. Overcoming these hurdles demands collaboration among researchers, clinicians, policymakers, and industry stakeholders to advance CKD diagnostics and therapeutics.

## 4. Therapeutic Targets of Renal Complications of Diabetes

The treatment of DKD involves a comprehensive approach aimed at managing diabetes by controlling blood sugar levels and blood pressure through lifestyle changes, medication, and, in some cases, insulin therapy as well as slowing the progression of kidney damage and preventing or managing complications. In addition, regular monitoring of kidney function through blood and urine tests is essential for early detection and intervention. In this section, we discuss the preclinically and clinically validated therapeutic approaches of DKD (Table 2).

### 4.1. Antihypertensive Drugs and RAAS Blockade

Antihypertensive drugs play a critical role in controlling blood pressure and slowing the progression of kidney damage in DKD. One of the key classes of antihypertensive medications used in DKD is drugs that target the renin–angiotensin–aldosterone system (RAAS) [148,149]. The RAAS is an important regulator of vascular resistance and blood volume, the major determinants of blood pressure. Hypertension is common in patients with diabetes and chronic activation of RAAS is shown to be involved in the development and progression of DKD [150]. In patients with DKD, optimal blood pressure control can slow the progression of kidney disease and prevent cardiovascular co-morbidities [151]. The drugs targeting RAAS in DKD include angiotensin-converting enzyme (ACE) inhibitors, angiotensin II receptor blockers (ARBs), and aldosterone antagonists. These medications block the effects of angiotensin II, a hormone that constricts blood vessels and increases blood pressure. By inhibiting angiotensin II, ACE inhibitors and ARBs dilate blood vessels, reduce blood pressure, and decrease intraglomerular pressure. This helps to protect the kidneys from further damage and slow the progression of DKD. The use of RAAS blockade in DKD is supported by extensive clinical evidence demonstrating their efficacy in reducing systemic blood pressure, delaying the progression of kidney disease and reducing the risk of adverse renal outcomes, such as ESRD and mortality. However, their effect on GFR decline has raised questions about potential benefits in advanced DKD [152]. The role of RAAS activation has also been identified in mitochondrial dysfunction which is a key event in the progression of DKD. Restoring mitochondrial function may be a potential therapeutic target in the early stages of DKD [149]. It has been demonstrated that with prolonged use of ACE inhibitors or ARBs, an “aldosterone breakthrough” may occur, in which aldosterone levels may rise to pre-treatment levels. Finerenone is a novel, nonsteroidal mineralocorticoid receptor antagonist (MRA) that has recently received regulatory approval with the indication of cardiorenal protection in patients with CKD associated with type 2 diabetes (T2D). Recent studies demonstrated a promising effect of finerenone in lowering the risk of CKD progression and CV events compared with placebo, with a minimal risk of hyperkalemia. Hence, finerenone may represent an important therapeutic option in DKD [153,154].

### 4.2. Glycaemic Control

Tight glycaemic control is essential in preventing or delaying the onset and progression of DKD. This involves monitoring blood glucose levels regularly and adhering to a treatment plan that may include lifestyle modifications, oral medications, insulin therapy, or other glucose-lowering medications as prescribed by a healthcare provider [155,156]. Major guidelines recommend lowering HbA1c to a goal ranging from <6.5% to <8.0% [157]. In patients with DKD, glycaemic targets need to be individualised. For type 1 diabetes (T1D), antihyperglycaemic treatment is based on daily insulin injections or the use of an insulin pump. On the other hand, for type 2 diabetes (T2D), glycaemic control is best achieved with a combination of lifestyle modifications (e.g., dietary restrictions, physical activity, and weight control) and pharmacologic therapy. Multiple medications are currently available to control diabetes. There are different classes of antihyperglycaemic agents that work with different mechanisms of action to lower blood glucose. Many of these have additional cardiorenal protective effects in addition to their hypoglycaemic effects [158]. Several glucose-lowering agents, including sodium–glucose cotransporter 2 (SGLT-2) inhibitors and glucagon-like peptide-1 receptor agonists (GLP-1 RA), have both renal and cardiovascular protective effects independent of their glucose-lowering effect. Currently, these agents are considered the mainstay of therapy for patients with T2D and DKD [159].

#### 4.2.1. Metformin

Metformin remains the preferred initial antihyperglycaemic therapy for most patients with T2D. Metformin lowers blood glucose levels primarily by decreasing the amount of glucose produced by the liver and also by increasing glucose utilisation in the skeletal muscle. Metformin is cheap and has proven efficacy with a low risk of hypoglycaemia. Limited data suggest the potential for risk of lactic acidosis in patients with lower eGFR [160]. For most patients with T2D and DKD, metformin and SGLT2 inhibitors are recommended as the first-line pharmacologic treatment with eGFR is above 30 mL/min/1.73 m^2^ [159]. In addition, metformin has been shown to activate muscle AMPK and promote glucose uptake, reduce ROS production, and delay the progression of DKD [161,162].

#### 4.2.2. SGLT2 Inhibitors

Initially developed as glucose-lowering drugs, SGLT2 inhibitors have been demonstrated to have significant renal and cardiovascular benefits. SGLT2 is involved in the reabsorption of glucose. A new class of medication, SGLT2 inhibitors, blocks this action, causing excess glucose to be eliminated in the urine. Bexagliflozin, canagliflozin, dapagliflozin, and empagliflozin are SGLT2 classical inhibitors that have been approved by the Food and Drug Administration (FDA) to treat type 2 diabetes. Several clinical studies showed significant cardiovascular and renal protective effects of SGLT2 inhibitors, and it has already become a milestone therapy for cardiorenal protection among patients with CKD [163]. The most common side effects include urinary tract infections, and genital yeast infections because of increased glucose excretion in the urine.

#### 4.2.3. Glucagon-Like Peptide-1 Receptor Agonist

GLP-1 RAs, or glucagon-like peptide-1 receptor agonists, are a class of injectable antihyperglycaemic medications used in the management of type 2 diabetes and obesity. These medications mimic the effects of GLP-1, a hormone produced in the intestines that stimulates insulin secretion, inhibits glucagon secretion, slows gastric emptying, and promotes satiety. These medications have shown benefits in lowering both blood glucose and body weight. Several GLP-1 RAs, including liraglutide, semaglutide, and dulaglutide, have little renal clearance and are safe to use even in patients with advanced-stage DKD [164]. GLP-1 receptor agonists appeared to be effective in reducing the risk of cardiovascular mortality, myocardial infarction, and stroke [165]. However, the addition of a GLP-1 analogue should be considered in patients with a contraindication or intolerance to metformin, in patients with a haemoglobin A1c greater than 1.5% over target, or in patients who do not reach their target A1c in three months, particularly in patients with atherosclerosis, heart failure, or chronic kidney disease [166]. The most common adverse effects of GLP-1 RAs include gastrointestinal symptoms, injection site reactions, mild tachycardia, and headaches.

#### 4.2.4. DPP-4 Inhibitors

Dipeptidyl peptidase-4 (DPP-4) inhibitors include sitagliptin, saxagliptin, linagliptin, alogliptin, and vildagliptin. These medicines increase insulin release from the pancreas in response to a meal and lower blood sugar with a low risk of causing hypoglycaemia and a neutral effect on body weight. These antihyperglycemic drugs can be given as a monotherapy to individuals who cannot tolerate the first-line medicine such as metformin, or they can be given in combination with other oral medicines depending on the blood sugar levels. Adverse effects of DPP-4 inhibitors include gastrointestinal symptoms, joint pain, and skin reactions. Concerns have been raised regarding the safety profile of DPP-4 inhibitors particularly in areas of pancreatitis and pancreatic cancer [167]. The major clinical trials using DPP-4 inhibitors to date have not shown significant effects on reducing the progression of DKD.

### 4.3. Agents Targeting Inflammation, Oxidative Stress and Fibrosis

Numerous studies have shown that low-grade inflammation is associated with the risk of developing diabetes and associated complications including DKD [168,169,170,171]. Hence, targeting pro-inflammatory molecules in DKD may offer a way for therapeutic interventions. However, the heterogeneity of the DKD patient population poses a challenge for clinical trials of novel anti-inflammatory therapies [172]. Finerenone, a non-steroidal selective mineralocorticoid receptor antagonist that induces natriuresis with reduced hyperkalaemia compared with steroidal antagonists, potentially provides anti-inflammatory and anti-fibrotic effects in DKD [153]. Finerenone inhibits MR over-activation and has recently been shown to slow the progression of CKD and reduce the risk of CV events in T2D [173]. In addition, preclinical studies have demonstrated the anti-inflammatory effects of SGLT2 inhibitors empagliflozin and dapagliflozin in association with a reduction in albuminuria and renal fibrosis in DKD [174,175]. The dysregulated activation of the complement system results in overt inflammation in many systemic diseases. Growing evidence indicates the activation of the complement cascade, has been implicated in the pathogenesis of diabetes and associated renal complications [176]. The anaphylatoxin C5a is a potent effector of complement-mediated inflammation. Excessive activation of the C5a-signalling axis promotes a potent inflammatory environment and is associated with mitochondrial dysfunction and an enhanced level of ROS production. Emerging preclinical evidence indicates that inhibition of the complement system may provide renoprotection in DKD by reducing inflammation and fibrosis while preserving the critical immunological defence functions of the complement system [177]. It appears that both C3a and C5a complements are likely to be key molecules that promote ROS generation and regulate immunometabolic pathways in the kidney, and both are suitable targets for interventions [178]. In a small cohort clinical trial, treatment with Avacopan, an inhibitor of the C5a receptor showed reduced proteinuria and MCP-1 levels in DKD [179]. Another potential anti-inflammatory compound pentoxifylline (PTF) is a methylxanthine phosphodiesterase inhibitor with anti-inflammatory and anti-fibrotic properties, resulting in the downregulation of TNF-α [180]. In a study of diabetic patients with nephropathy, PTF reduced the level of albuminuria, displaying renoprotective properties [181].

Antioxidative agents such as NRF2 activators have attracted interest for their potential to mitigate oxidative stress, inflammation, and kidney damage in DKD [182,183]. Preclinical studies have demonstrated that NRF2 activation can mitigate kidney injury and fibrosis in animal models of DKD. NRF2 activators have been shown to preserve kidney function, reduce proteinuria, attenuate glomerular and tubulointerstitial damage, and inhibit the progression of DKD [156]. Several natural compounds and synthetic molecules have been identified as NRF2 activators, including sulforaphane (found in cruciferous vegetables), bardoxolone methyl, and dimethyl fumarate. One of the most notable clinical trials involving bardoxolone methyl in DKD was the BEACON trial. The BEACON trial was a phase 3, randomised, double-blind, placebo-controlled study designed to assess the effects of bardoxolone methyl on kidney function in patients with DKD and type 2 diabetes [184]. The BEACON trial was prematurely terminated due to safety concerns. An interim analysis revealed a higher incidence of cardiovascular events, particularly heart failure, in patients receiving bardoxolone methyl compared to those receiving placebo. As a result, the trial was stopped early, and further development of bardoxolone methyl for DKD was halted [185]. Curcumin is another important NRF2 activator that plays a role in decreasing oxidative stress [186]. Curcumin decreased expression of MCP-1, IL-1β, and TNF-α leading to anti-inflammatory responses in type 2 diabetic patients [187,188]. While NRF2 activation holds potential as a therapeutic strategy for DKD, further research is needed to elucidate the optimal timing, duration, and dosing of NRF2 activators, as well as their long-term safety profile.

Antifibrotic treatments for DKD aim to inhibit or reverse the fibrotic processes that contribute to the progression of kidney damage and decline in renal function. Fibrosis, characterised by excessive deposition of extracellular matrix proteins such as collagen, is a hallmark of DKD and can lead to renal scarring and dysfunction. Inhibitors of TGF-β signalling, such as pirfenidone and halofuginone, have been studied for their antifibrotic effects in DKD. These agents may interfere with TGF-β-mediated fibrotic pathways and reduce the accumulation of extracellular matrix proteins in the kidney [189,190,191]. Connective tissue growth factor (CTGF) is another profibrotic factor implicated in DKD. Inhibition of CTGF signalling may attenuate renal fibrosis and preserve kidney function. Monoclonal antibodies targeting CTGF or small molecule inhibitors of CTGF have shown promise in preclinical studies [192,193]. Various agents targeting the extracellular matrix, such as lysyl oxidases (LOXs) inhibitors and matrix metalloproteinase inhibitors, have been investigated for their potential antifibrotic effects in DKD. These agents may disrupt the synthesis, cross-linking, or degradation of extracellular matrix proteins and prevent the development of renal fibrosis [194]. While these anti-inflammatory and antifibrotic approaches show promise in preclinical studies, their efficacy and safety in humans with DKD require further investigation in well-designed clinical trials. Combination therapies targeting multiple pathways involved in renal inflammation and fibrosis may offer synergistic benefits and improve outcomes for individuals with DKD.

## 5. Conclusions

Individuals with diabetes are at increased risk of developing DKD, which can progress to ESRD and necessitate dialysis or kidney transplantation. Current therapies for DKD primarily focus on glycaemic control, blood pressure management, and the use of medications that target inflammation and fibrosis. While these treatments have demonstrated some level of benefit in reducing the progression of DKD and preserving kidney function, there is still a need for more targeted and effective therapies. Identifying biomarkers and novel mechanism-based specific targets is crucial for developing precision medicine approaches to the treatment and prevention of DKD. Biomarkers can help in early detection, risk stratification, and monitoring of disease progression, while specific targets can be exploited to develop pharmacological agents that address the underlying pathophysiology of DKD more effectively.

## Figures and Tables

**Table 1:** Biomarkers of DKD and their key characteristics for risk prediction and diagnosis.

Biomarker	Key Characteristics
Albumin	Gold standard marker of early detection of kidney damage and positively correlated with DKD progression.
Glycated albumin	Glycated albumin offers short-term measurement of glucose levels in serum. HbA1c can be used as a predictor of diabetes and associated complications and monitoring of the disease.
Transferrin	Early and sensitive diagnostic marker of DKD.
Adiponectin	Low serum and high urine levels are used as risk predictors in DKD.
Ceruloplasmin	Elevated in diabetic patients and track glycaemic index and inflammation.
Laminin	Urinary laminin fragments can reflect early features of DKD such as mesangial expansion, ECM accumulation and renal injury.
Podocyte number and podocyte-specific proteins	Urinary podocyte loss could predict DKD initiation and progression.
NGAL	Potential biomarker to predict disease severity and progression.
NAG	Increases in urine with tubular injury. Found in high concentrations in diabetic normoalbuminuric patients. Could serves as a marker for treatment efficacy and prognosis of disease.
KIM-1	Involved in phagocytosis of fatty acids by proximal tubule cells. Sensitive marker of AKI and chronic kidney disease progression.
α1- and β2-Microglobulin	Under normal conditions are almost completely reabsorbed by proximal tubule cells. Increased in urine with decline renal function.
Fatty acid binding proteins	Involved in tubular injury. Urinary excretion of L-FABP and H-FABP are observed in DKD. L-FABP may be an early biomarker and potential therapeutic target.
RBP4	Elevated serum levels in insulin resistance and type 2 diabetes.
Pro-Inflammatory markers	Elevated levels in plasma and urine may indicate intrarenal inflammation in CKD.
Oxidative stress markers	Elevated levels in plasma and urine may predict early stage of CKD.
Omics approaches	Useful in early detection and risk prediction of CKD but expensive.

**Table 2:** Mechanism of action, adverse effects and benefits of DKD therapeutic targets.

Drugs	Proposed Targets/Mechanisms	Adverse Effects	Over all Benefits and Remarks
**Antihypertensive drugs and RAAS Blockade**			
Angiotensin-converting enzyme (ACE) inhibitors	Inhibits ACE activity and production of angiotensin II	Cough, hyperkalaemia, angioedema, acute kidney injury	Dilate blood vessels, reduce blood pressure, and decrease the intraglomerular pressure reducing systemic blood pressure and delaying the progression of kidney disease by reduction in ROS and inflammation
Angiotensin II receptor blockers (ARBs)	Inhibits binding of angiotensin II to the receptor	Hyperkalaemia, acute kidney injury	
Aldosterone antagonists	Mineralocorticoid receptor antagonists	Hyperkalaemia	
**Glycaemic controls**			
Insulin	Stimulates the uptake of glucose by cells	Risk of hypoglycaemia, sweating, anxiety	Maintain carbohydrate, protein and lipid metabolism. First-line treatment of type 1 diabetes mellitus
Metformin	Decreases the amount of glucose produced by the liver and increases glucose utilisation in the skeletal muscle	Gastrointestinal symptoms, risk of lactic acidosis	Cheap, low risk of hypoglycaemia, activate muscle AMPK and promotes glucose uptake, reduce ROS production, and delay progression of DKD
Sodium–glucose cotransporter 2 (SGLT-2) inhibitors	Blocks reabsorption of glucose by inhibiting SGLT-2, causing excess glycosuria	Urinary tract infections, genital yeast infections	Cardiovascular and renal protective effects
Glucagon-like peptide-1 receptor agonist (GLP-1 RA)	Stimulates insulin secretion, inhibits glucagon secretion, slows gastric emptying, and promotes satiety.	Gastrointestinal symptoms, injection site reactions, mild tachycardia, pancreatitis and headaches	Lowers blood glucose and body weight. Reduces the risk of cardiovascular mortality, myocardial infarction, and stroke
DPP-4 inhibitors	Increases insulin release from the pancreas	Gastrointestinal symptoms, joint pain, and skin reactions	A low risk of causing hypoglycaemia and a neutral effect on body weight, safety concerns in regard to pancreatitis and pancreatic cancer
**Agents targeting inflammation, oxidative stress and fibrosis**			
Finerenone	Inhibits the effects of mineralocorticoids like aldosterone and cortisol	Hyperkalaemia	Slow the progression of CKD and reduce the risk of CV
Avacopan	An inhibitor of C5a receptor	Nausea, vomiting, diarrhoea, headache.	Reduced proteinuria and MCP-1 level in DKD
Pentoxifylline (PTF)	Phosphodiesterase inhibitor with anti-inflammatory and anti-fibrotic properties		reduced the level of albuminuria, displaying renoprotective properties
NRF2 activators: Sulforaphane, bardoxolone methyl, and dimethyl fumarate Curcumin	Enhances NRF2 activity and antioxidant defence mechanism Causes anti-inflammatory responses	Increased CVD risk and morality	Mitigate kidney injury and fibrosis in animal models of DKD. Unstable molecular structure, limited absorption and faster degradation and elimination
Pirfenidone and Halofuginone	TGF-β-mediated fibrotic pathways and reduce the accumulation of ECM proteins in the kidney	Photosensitivity and rash, gastrointestinal disorders	Contraindicated in persons undergoing dialysis.

## Data Availability

Not applicable.
